# Not So Benign: Revisiting Pure Membranous Lupus Nephritis

**DOI:** 10.3390/jpm15120580

**Published:** 2025-11-30

**Authors:** Martina Uzzo, Marta Calatroni, Gabriella Luisa Moroni

**Affiliations:** 1 Nephrology and Dialysis Division, IRCCS Humanitas Research Hospital, Via Manzoni 56, Rozzano, 20089 Milan, Italy; marta.calatroni@hunimed.eu (M.C.); gabriella.moroni@hunimed.eu (G.L.M.); 2 Department of Biomedical Sciences, Humanitas University, Via Rita Levi Montalcini 4, Pieve Emanuele, 20072 Milan, Italy

**Keywords:** lupus nephritis, histology, kidney biopsy, classification

## Abstract

Pure membranous lupus nephritis (pMLN, ISN/RPS-class V) is a rare form of lupus nephritis (LN). Despite being associated with significant comorbidities, it has traditionally been considered a less aggressive subtype. Emerging data challenges this perception, highlighting its potential for chronic kidney disease progression and kidney failure. pMLN is pathologically defined by subepithelial immune-complex deposits and typically presents with nephrotic syndrome, preserved renal function, and fewer systemic/immunologic manifestations compared to proliferative LN (ISN/RPS-classes III/IV). Repeat biopsies reveal frequent histological class switching from pMLN to proliferative and mixed LN forms, underscoring the dynamic nature of the disease and the limitations of clinical markers in reflecting histological activity. While the ISN/RPS kidney biopsy classification provides important prognostic insight, it does not fully capture underlying molecular heterogeneity. Recent advances in precision medicine, including proteomic and biomarker studies (e.g., EXT1/2, NCAM1), offer promising tools for patient stratification and tailored treatments. International guidelines now recommend immunosuppressive therapy for pMLN, aligning treatment strategies more closely with those for proliferative and mixed LN. Overall, pMLN should be considered a distinct but clinically relevant LN subtype requiring personalized management based on clinical, histological and molecular features. Long-term monitoring is essential, as baseline presentation does not reliably predict treatment response or disease trajectory.

## 1. The Multiple Facades of Lupus Nephritis

### 1.1. General Overview

Systemic Lupus Erythematosus (SLE) is a chronic autoimmune disease characterized by autoantibody production and immune complex deposition, triggering inflammation and organ damage [1]. Kidney involvement in SLE is formally called lupus nephritis (LN): 40–60% of SLE patients in different case series develop LN within 5 years from disease onset, and this is responsible for a large amount of SLE-related mortality and morbidity [2,3,4]. Ethnicity influences the risk of developing LN, with Black, Hispanic, and Asian patients with SLE being more likely to experience LN during the progression of the disease compared to White patients. Additionally, Black and Hispanic individuals often exhibit more severe clinical and histopathological features at diagnosis compared to White patients [5]. Clinical presentation varies from isolated microhematuria to nephrotic syndrome and/or acute decline of kidney function, without a clear clinico-pathological correlation [6,7]. For these reasons, to assess the therapy and the prognosis, a kidney biopsy is mandatory. Untreated moderate/severe forms on clinical and/or histological basis have an unfavorable course, leading to kidney insufficiency: risk factors for progressive kidney disease are Black ethnicity, male sex, high serum creatinine, and nephrotic proteinuria (>4 g/day) at diagnosis, incomplete/no remission after induction therapy and frequent relapses—particularly nephritic flares characterized by deterioration of renal function [8,9,10].

Since the introduction of the 2012 SLICC classification criteria for SLE, the combination of kidney biopsy findings consistent with LN and the presence of ANA or anti–double-stranded DNA antibodies has been considered to classify a patient with SLE [11]. Despite ongoing debate on criteria, renal involvement was confirmed as a key feature in the latest 2019 SLE EULAR-ACR classification, underscoring the central role of the kidney in disease expression and severity [12].

### 1.2. The Central Role of the Histopathological Classification

LN exhibits a wide range of histopathological features, which can sometimes resemble other forms of immune complex-mediated glomerulonephritis. However, certain findings are particularly indicative of LN. A hallmark is the “full house” immunofluorescence staining pattern, marked by dominant IgG and co-deposits of IgA, IgM, C3, and C1q. While this pattern is most characteristic of LN, it can occasionally appear in other conditions grouped into the definition of non-lupus full house nephropathy [13]. Another supportive finding is the presence of tubuloreticular inclusions by electron microscopy within glomerular endothelial cells; they are rare, but virtually pathognomonic, although they are also linked to alpha-interferon activity. LN typically features immune deposits across various glomerular regions—mesangial, subendothelial, and subepithelial—as well as in extraglomerular sites such as tubular basement membranes, the interstitium, and blood vessels [11,14,15]. However, despite the importance of immunofluorescence, until recently, the histological classifications of LN were based entirely on light microscopy [16].

Over time, LN histopathological classification has changed dramatically in an attempt to standardize definitions, highlight clinically significant lesions, and enhance interobserver consistency, reflecting the advances in the understanding of LN pathophysiology [16]. The World Health Organization (WHO) morphologic classification, introduced in 1974 and updated in 1982 and 1995, first stratified LN into five classes based on light microscopic findings of glomerular lesions: membranous lupus nephritis was classified as class V [17]. In the 1982 updated WHO classification, class V was further divided into four subclasses (Va to Vd), according to the presence of additional mesangial involvement (Vb) or additional proliferative changes (Vc, Vd). This framework lacked uniformity in defining mixed forms of LN, which were all classified as class V [16,18,19]. The International Society of Nephrology/Renal Pathology Society (ISN/RPS) classification, published in 2003 and revised in 2018, refined this system by incorporating additional features from immunofluorescence and electron microscopy (Table 1) [6,16].

**Figure 1:**
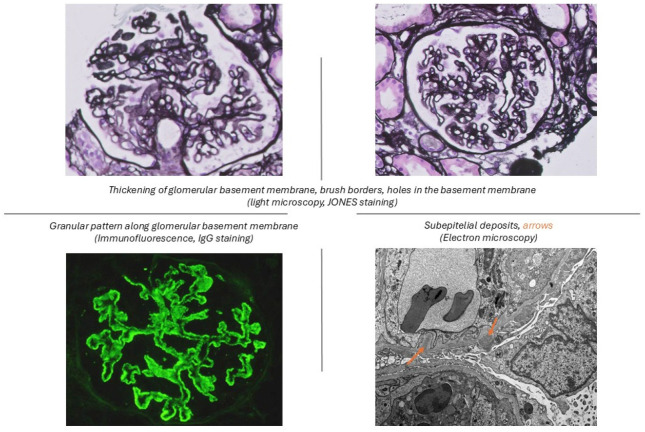
Histopathology features of membranous lupus nephritis.

In particular, Vc and Vd were reclassified into mixed categories (Classes III + V or IV + V) based on the extent of proliferative features (class III—focal, <50% glomeruli; class IV—diffuse, ≥50% glomeruli)**.** This reclassification eliminated ambiguities related to mixed patterns and emphasized clinical relevance. The ISN/RPS [6] class V was defined as a membranous lupus nephritis (MLN) with global or segmental continuous granular subepithelial immune deposits independent of the presence or absence of mesangial involvement (Figure 1).

If a distributed membranous lesion (involving more than 50% of the tuft in over 50% of the glomeruli, as identified by light microscopy or immunofluorescence) is found alongside an active lesion of class III or IV, both conditions should be included in the diagnostic report [6,16]. The prevalence of histological classes has remained relatively stable over the years, with Class IV accounting for approximately 50%, Class III for 25%, and Class V for 20%. However, the proportion of mixed forms (III + IV and IV + V) has increased over time, ranging from 35–54% of MLN cases in different studies [18,21,22], likely reflecting updates in the classification criteria [23].

Moreover, the ISN/RPS classification [6] first incorporated the activity and chronicity indices into biopsy reports, grading the individual morphologic components as a guide for treatment. While the chronicity score—which identifies chronic, irreversible lesions that are not responsive to therapy—is a very important prognostic tool in all histological classes, the activity index is of great value for assessing the therapeutic strategy for LN proliferative forms only (ISN/RPS-classes III/IV) (Table 2) [15,16].

The ISN/RPS [6] LN classification is an important tool to order different kinds of kidney involvement in LN, providing prognostic information on renal outcomes and enabling standardized comparisons across studies. Whilst it does not reflect the pathogenetic mechanisms hidden behind the lesions, the ISN/RPS [6] LN classification can guide therapeutic decisions, as suggested by major international recommendations [24,25,26]. Altogether, Classes III and IV, which are characterized by intense glomerular inflammation, endocapillary hypercellularity, and crescent formation, are considered the most severe forms of LN. They require more aggressive therapy to avoid the progression to chronic kidney disease and kidney failure. In contrast, pure MLN (pMLN, ISN/RPS-class V) is defined by primary and exclusive podocyte injury, which manifests as proteinuria. This explains why pMLN causes less acute damage compared to the inflammatory processes observed in proliferative forms.

### 1.3. Dynamic Class Switching in Repeat Biopsies

During the follow-up, several factors (i.e., the patient compliance, the incomplete response to treatment or reactivation of disease, the presence of comorbidities) determine histological changes: in difficult cases, repeat kidney biopsies can provide new valuable insights into disease progression and help assess treatment effectiveness [5,27,28]. In a multicenter cohort of 92 LN patients, 91.3% of whom were of Caucasian ethnicity, a repeat kidney biopsy was performed for clinical reasons (persistent proteinuria, nephritic or nephrotic flares) after a mean of 6.7 ± 4.9 years from the baseline biopsy. Class switches between the first and second biopsies occurred in 50.5% of cases, mainly from Class V LN to proliferative classes. Twenty-five patients (27.2%) developed end-stage kidney disease (ESKD) within nine months after the second kidney biopsy. While none of the clinical or histological variables at the first biopsy were associated with ESKD occurrence, the activity index, chronicity index, and proteinuria at the second biopsy were independent predictors of ESKD in multivariable analysis [29].

In a recent study, repeated kidney biopsies were performed on clinical indication in patients with persistent proteinuria or unexplained decline of renal function, after 3.9 (0.3–28.0) years after the baseline kidney biopsy. Among 220 patients (542 biopsies)—mostly African American (54%) or Caucasian (27%)—one third experienced a switch in ISN/RPS [6] LN classes. Conversion from pMLN to proliferative LN occurred in 41% of cases. Of note, pMLN in a repeat biopsy was preceded by pure proliferative/mixed LN in 52% of cases, and two years after the second biopsy, the evidence of prior proliferative/mixed LN in pMLN was associated with a higher risk of kidney failure [30].

Beyond repeat biopsies performed on a clinical indication, the potential role of protocol biopsies carried out 12 months after the start of induction therapy is under evaluation by the ReBioLup trial (NCT04449991). One of the trial’s main objectives is to assess the prognostic value of repeat biopsy in pMLN for long-term renal outcome. If successful, this study could provide robust evidence for the integration of protocol biopsies into clinical practice.

## 2. Pure Membranous Lupus Nephritis

### 2.1. Introduction to Pure Membranous Lupus Nephritis

pMLN accounts for 15 to 20% of cases of biopsy-proven LN and typically presents clinically with nephrotic syndrome, similar to the idiopathic membranous nephropathy, and in contrast to the more aggressive proliferative LN classes, which are mostly characterized clinically by deterioration of kidney function, active urinary sediment, and a variable amount of proteinuria [21]. Despite being associated with a high rate of significant comorbidities, pMLN is traditionally regarded as having a low rate of progression to ESKD. However, recent studies have highlighted the potential for pMLN to progress to chronic kidney disease [19,31]. Moreover, until recently, the suggested treatment approach in pMLN also seemed to be different from other ISN/RPS [6] classes, especially in cases with sub-nephrotic proteinuria [26].

This highlights the importance of recognizing pMLN as a distinct entity, with specific clinical, pathological features and long-term outcomes, which will be discussed in detail in the following paragraphs.

### 2.2. Clinical Phenotype of Pure Membranous Lupus Nephritis

Around fifty percent of patients with pMLN present with nephrotic syndrome (55–64% in different studies [19,21,32]), characterized by heavy proteinuria, hypoalbuminemia, hyperlipidemia, and edema [31,32]. Kidney function is often preserved at the time of diagnosis, although some patients may experience functional renal dysfunction secondary to anasarca. Of note, pMLN patients may lack extrarenal symptoms at onset (i.e., fever, lymph node, skin, joint, serous or central nervous system involvement) and present without any serological manifestation of SLE (i.e., complement levels may be normal, and anti-dsDNA antibodies or C1q may be in normal range), presenting only with proteinuria, which can precede other clinical or serological features for years [19,21,32,33]. Unlike proliferative LN, pMLN does not exhibit intense glomerular inflammation, endocapillary hypercellularity, or crescent formation. The absence of proliferative activity is associated with a milder clinical presentation, typically without nephritic syndrome or rapidly progressive kidney dysfunction. Accordingly, pMLN rarely presents with significant hematuria or hypertension at onset, supporting its characterization as a less aggressive disease phenotype [31,34].

However, patients with pMLN are at increased risk for cardiovascular disease and thrombotic complications, exacerbated by hypoalbuminemia, glucocorticosteroid (GC) use, and specific immunosuppressive therapy. Nephrotic syndrome leads to hypercoagulability, dyslipidemia, and a higher risk of infection. Elevated cholesterol and triglycerides frequently accompany proteinuria and hypoalbuminemia or prolonged corticosteroid treatment, with LDL- and VLDL-cholesterol levels being particularly predictive of atherosclerosis. Patients with nephrotic syndrome face a heightened risk of thrombotic events due to elevated fibrinogen, low albumin, urinary loss of anticoagulants (i.e., protein C, protein S, antithrombin III), and hyper viscosity linked to hypercholesterolemia. Additionally, antiphospholipid antibodies, present in around 20% of patients, further predispose patients to arterial and venous thrombosis. Hypertension, commonly reported in SLE, contributes to accelerated atherosclerosis, cardiovascular disease, and kidney damage. GCs can also promote insulin resistance and type 2 diabetes, further increasing cardiovascular disease risk [35].

### 2.3. Pathogenesis: How Membranous Lupus Nephritis Leads to Kidney Injury

The pathogenesis of the membranous involvement in LN (MLN, including both pure and mixed proliferative forms) and the eventual differences from other forms of LN and primary membranous nephropathy remain poorly understood. Immune and inflammatory responses in experimental models of membranous LN mirror mechanisms seen in proliferative LN, but MLN-specific research is limited [36].

MLN pathogenesis involves complex immune-mediated mechanisms, primarily driven by the production of autoantibodies against nuclear and cellular antigens, leading to the deposition of subepithelial immune deposits. The immune complexes, formed either in situ or deposited from circulation, trigger complement activation and membrane attack complex (MAC) formation, leading to intrarenal inflammation and podocyte injury. The importance of MAC was demonstrated in animal models of MLN, where deficiency of C6 (part of MAC C5b-9) prevented proteinuria without affecting subepithelial immune deposit formation [37].

Subepithelial deposits are the histological counterpart of primary membranous nephritis, though the pathogenetic mechanisms seem to be different. Primary membranous nephritis is characterized by in situ immunocomplex deposition, due to autoantibodies targeting specific epitopes on podocytes (i.e., PLA2R, mainly IgG4) [38]. In contrast, a multiplicity of antibodies directed to exposed autoantigens is involved in LN, leading to in situ immunocomplex formation, circulating immunocomplex deposition, and deposit of histone-rich nucleosomes. In this context, it remains unclear why, in MLN, these immune complexes are selectively deposited in the subepithelial area [39]. Autoantibodies in MLN (mainly IgG1, IgG2, IgG3) activate the classical complement pathway, contributing to podocyte injury and proteinuria. Unlike in primary membranous nephritis, where complement activation is limited, both the classical and alternative pathways play a role in MLN [40].

### 2.4. Towards Precision Medicine: Biomarkers in Membranous Lupus Nephritis

Precision medicine aims to align specific treatments with the underlying molecular pathways of specific patients’ subgroups, thereby enhancing therapeutic efficacy, safety and cost-effectiveness. This approach is particularly valuable in complex autoimmune diseases, such as SLE, as it predicts disease risk, facilitates earlier intervention, and reduces unnecessary exposure to potentially harmful therapies [41,42].

Many efforts are being made to identify early indicators of response in LN; however, only a few may be specifically applied to MLN [38,43,44,45]. In 2015, Moroni et al. showed that the presence of anti-C1q was the most reliable serological marker in differentiating proliferative LN (ISN/RPS [6] class III/IV) vs. non-proliferative LN (ISN/RPS [6] class II/V), as it is more frequently detected in the first compared to the latter group [45]. These results were recently confirmed in a larger cohort study [46].

New biomarker strategies include the analysis of serum, urine, and other human tissues using advanced technologies such as transcriptomics (microarray profiling, RNA-seq, machine learning), genetic and epigenetic profiling (DNA methylation, histone modifications, microRNAs), and immune profiling (cell and mass cytometry targeting the innate and adaptive immune systems).

In a recent study, Fava et al. explored the proteomic signature of ISN/RPS [6] LN classes in urine to identify non-invasive biomarkers of intrarenal activity. Patients with pure proliferative and mixed LN demonstrated similar proteomic profiles, reflecting shared biological processes such as innate immune activation, neutrophil degranulation, viral life cycle pathways, and extracellular protease activity, consistent with their similar clinical presentation and prognosis. The proteomic signature in pMLN was largely shared with proliferative LN, indicating overlapping core pathways across proliferative, mixed, and membranous LN. However, many proteins characteristic of proliferative LN (pure or mixed) were absent in pMLN, highlighting distinct biological mechanisms specific to proliferative LN [43].

In addition to the existing histopathological classification, novel biomarkers reflecting molecular mechanisms of MLN pathology have been identified. A subset of potential antigens, including exostosin 1 (EXT1) and EXT2, as well as neural cell adhesion molecule 1 (NCAM1), has been identified as candidates in secondary membranous nephropathy associated with autoimmune diseases, including MLN [38,47].

EXT1 and EXT2 are intracellular proteins, members of the exostosin glycosyltransferase family, which regulate the synthesis of the heparan sulfate backbone via chain elongation and multiple signaling pathways; the loss of their function causes hereditary multiple osteochondromas. However, their specific role in MLN still needs to be clarified [38,44].

In a recent cohort of 283 MLN patients—with or without proliferative changes—one third were positive for EXT1/2, with a higher prevalence of pure class V versus mixed forms (44.2% vs. 19.4%, *p* < 0.001). Kidney biopsy specimens were incubated with rabbit anti-EXT1; EXT1/2 positivity was defined by the presence of a granular staining for both EXT1 and EXT2 along the glomerular basement membrane. Patients with positive EXT1/EXT2 showed significantly lower disease activity both in terms of clinical involvement (SLEDAI score: 12 vs. 14, *p* = 0.015) and lab tests (lower creatinine: 57 vs. 73 umol/L, *p* < 0.001; higher hemoglobin: 114 g/L vs. 106 umol/L, *p* = 0.006), and a lower amount of both active and chronic features on kidney biopsy. This result may be partially explained by a selection bias: of the EXT1/EXT2-positive patients, 60.2% were categorized as pure class V, whereas most of EXT1/2-negative patients (68.5%) were classified as class V + III/IV. After adjusting for age, gender, serum creatinine, and proteinuria, EXT1/EXT2-positive patients were less likely to develop adverse renal outcomes compared to EXT1/EXT2-negative patients, but the difference disappeared after the inclusion of the chronicity index among the variables, suggesting that these findings may reflect disease chronicity rather than distinct pathogenetic mechanisms [48]. In a previous study including 374 patients, 39 with pMLN were tested for EXT1/2: EXT1/2-positive patients presented with a lower serum albumin (22.8 5.7 vs. 29.1 7.7 g/L, *p* = 0.016) and a tendency towards higher proteinuria. The same results were obtained when evaluating a combined cohort of pure and mixed MLN [49]. Other authors obtained similar results [50,51].

Another biomarker under investigation for MLN is Neural Cell Adhesion Molecule 1 (NCAM1), a member of the Ig superfamily, which is only slightly expressed in healthy kidneys [52]. NCAM1 was found to be a target antigen for MLN: it co-localizes with IgG in glomerular immune deposits, including the subepithelial region, accounting for 6.6% of all cases of MLN [52]. In a recent cohort of 361 patients with MLN, NCAM1 positivity was observed in 5% of cases (18 patients), distributed equally between pure and mixed forms. Interestingly, although there were no differences in response to therapy and ESKD, NCAM1-positive patients had a markedly higher risk of death compared to NCAM1-negative patients (27.8% vs. 8.1%; *p* = 0.007) [53].

Both EXT1/EXT2 and NCAM1 are associated with subepithelial immune deposits, suggesting mechanisms of injury in MLN that are different from other forms of membranous nephropathy [49,52]. They may be regarded as the first histological biomarkers of MLN; however, these preliminary data warrant further validation in larger cohorts before their introduction in clinical practice.

### 2.5. Long-Term Renal Outcomes in Membranous Lupus Nephritis: Risk Factors and Survival

Several studies have examined the long-term outcomes and prognosis of pMLN; the main ones published after 2000 are summarized in Table 3 [2,19,31,54,55,56,57,58,59,60].

Reported 10-year renal survival rates vary widely between studies, ranging from 47% to 90% [19,31]. Key prognostic factors for kidney survival include baseline serum creatinine, the chronicity index, and the achievement of complete remission within the first six months of treatment; moreover, patients who fail to achieve remission are at significantly higher risk for progression to kidney failure [31]. In a pMLN cohort of 66 patients, Mercadal et al. showed a renal survival at 10 years of 88%; thrombosis and initial low hemoglobin levels were independent predictors of poor outcomes [19]. Later, Mok et al. tested the efficacy of combined prednisone and azathioprine therapy in a small cohort of 38 pMLN patients: 67% achieved complete remission, and only 19% experienced relapses over a mean follow-up of 90 months [32]. During 6–7 years of follow-up, the relapse rate has been reported to range from 19% to 49% [3,19,57,59]. Similar to kidney survival, mortality varies between studies, mostly ranging from 2% to 6% in recent cohorts after an observation of 5–7 years, with an exceptionally high rate of 33% reported in an older, smaller study of 33 patients followed for 5 years [58]. The high outcome variability across studies likely reflects the heterogeneity of study periods, populations and treatment strategies. Earlier studies reported higher mortality, largely due to less aggressive treatment approaches historically applied to pure MLN forms and the limited availability of effective immunosuppressive or targeted therapies at that time [19,54].

### 2.6. Comparative Outcomes: Pure Membranous vs. Proliferative/Mixed Lupus Nephritis

Only a few studies have specifically compared pure MLN with mixed or purely proliferative forms, and those available are limited by small sample sizes and short-term follow-up. The design and main findings of the studies are summarized in Table 4.

Sloan et al. stratified patients into subcategories based on the WHO classification, Patients with pMLN and MLN with mesangial involvement (WHO Va/Vb) had significantly better renal survival than those with proliferative forms (WHO Vc/Vd) [17], with 5-year and 10-year renal survival rates of 72% for WHO Va/Vb and of 48–20% for WHO Vc and Vd [16,18]. In a larger cohort of 103 MLN patients, 67 were classified as pMLN and 36 as mixed forms according to the ISN/RPS 2003 classification, Moroni et al. investigated clinical, histopathology and laboratory features at presentation and long-term prognosis: those with mixed forms had more frequent nephrotic syndrome (66.6% vs. 44.7%, *p* < 0.05), lower C3 and C4, higher anti-DNA positivity and worst kidney function (eGFR 93 vs. 112 mL/min, *p* < 0.05) at presentation compared to pMLN patients; moreover, mixed MLN had significantly higher activity and chronicity indexes. Fifty percent of the mixed LN patients were in remission 17 months after the beginning of therapy, whereas it took 3 years to achieve remission in 50% of the patients with pMLN (*p* < 0.05). However, no differences in terms of achievement of kidney remission (94.5 vs. 94.0%) and kidney survival (85.8 vs. 86.0%) at 10 years were detected between groups [21]. In a study involving 260 LN patients, Farinha et al. compared the pMLN subgroup (203 patients) versus the pure proliferative LN one (47 patients): patients with pMLN exhibited nearly normal complement levels and less frequent positivity for anti-dsDNA antibodies compared to pure proliferative forms; similar results had been previously obtained by the same study group on a smaller cohort of 187 patients [62]. Short- and long-term kidney outcomes were similar between groups [61]. Kharouf et al. recently focused on two slightly different subgroups: 51 patients with pMLN were compared to 164 patients with mixed (44 patients) or pure PLN (120 patients), who were followed for a median time of 8 years. pMLN patients showed higher baseline eGFR (103.3 vs. 87 mL/min/1.73 m^2^, *p* < 0.05) and lower chronicity index at kidney biopsy compared to the others. Short-term outcomes were similar between groups in this cohort as well. For long-term outcomes, proliferative LN was associated with worse renal and non-renal outcomes, but the difference was not statistically significant [i.e., 4 (7.8%) patients had kidney failure in pMLN vs. 24 (14.7%) in mixed/pure proliferative LN; *p* = 0.30]; of note, the resolution of proteinuria was slightly—though insignificantly—slower in the pMLN group compared to the mixed form and the proliferative LN groups [22].

While pMLN generally presents better baseline renal function and lower chronicity indices than proliferative LN, recent studies have shown that long-term outcomes, including CKD progression and kidney failure, are comparable between the two groups. Despite differences in initial presentation, pMLN is not entirely benign, emphasizing the need for long-term monitoring and individualized treatment.

### 2.7. Therapeutic Strategies for Membranous Lupus Nephritis: From Guidelines to Clinical Practice

Therapy for MLN aims to preserve kidney function, to prevent complications—particularly in patients with nephrotic syndrome—and to simultaneously control the underlying immunologic activity to resolve kidney and systemic inflammation.

All patients with SLE, regardless of the degree and type of disease activity, should receive treatment with hydroxychloroquine (5 mg/kg/day) unless contraindicated; this is valid for pMLN as well [26]. Additionally, general supportive measures focused on preserving kidney function should be used (i.e., dietary sodium restriction, blood pressure control, minimization of proteinuria with renin-angiotensin system inhibition, and treatment of dyslipidemia). Anticoagulant therapy is recommended in hypoalbuminemia-driven hypercoagulability states [35]. Moreover, in patients who achieved stable remission and had mild deterioration of renal function, sodium-glucose cotransporter 2 inhibitors may also be of benefit for proteinuria reduction and kidney function long-term preservation, although studies focusing on MLN are lacking [63].

Patients with active class III or IV LN, including those with a membranous component, are typically treated with standard therapy for proliferative LN [64,65,66]. However, treatment strategies for pMLN remain less well-defined, with only one small, randomized trial specifically addressing this subgroup [33]. Consequently, decisions regarding the use of immunosuppressive therapy are based on lower-quality evidence, including expert clinical opinion [24,26].

**Figure 2:**
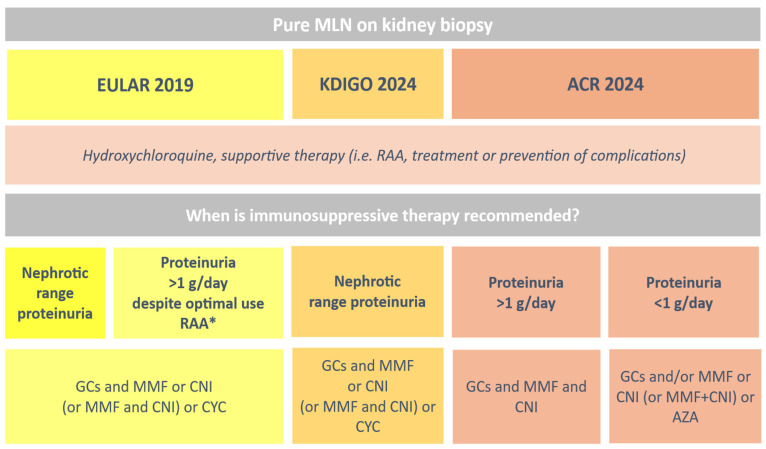
The use of immunosuppressive therapy in pure Membranous Lupus Nephritis (pMLN). RAA, Renin-Angiotensin–Aldosterone system blockers; GCs, Glucocorticoids; MMF, Mycophenolate Mofetil; CYC, Cyclophosphamide; AZA, Azathioprine; CNI, Calcineurin Inhibitor; * for a reasonable time period (e.g., at least 3 months).

Oral or intravenous pulse corticosteroids represent the cornerstone of treatment for all active forms of LN, including pMLN, although the optimal dosing and duration of therapy remain debated. In contrast, the use of immunosuppressive agents, particularly in pure membranous forms, is less well established (Figure 2).

The recommendation to use immunosuppressive therapy in patients with pMLN and proteinuria exceeding 1 g/day was first introduced in the 2019 EULAR guidelines [64]. More recently, the updated 2024 KDIGO guidelines on lupus nephritis recommend a tailored approach to manage patients with pMLN based on their level of proteinuria. Patients with nephrotic-range proteinuria should be treated with immunosuppressive therapy (induction regimen plus steroids), while those with lower levels of proteinuria may be managed with supportive therapy only. Hydroxychloroquine should be added on top of therapy in both groups [65]. A small randomized controlled trial showed that remission was more likely with prednisone plus cyclophosphamide (60%) or prednisone plus cyclosporine (84%) compared to prednisone alone (27%) in patients with pMLN and nephrotic syndrome. Cyclophosphamide also maintained remission longer, with no relapses within a year, while 40% of cyclosporine-treated patients relapsed within a year after sudden discontinuation of cyclosporine [33]. To date, the study by Austin et al. remains the only randomized controlled trial specifically focused on pMLN, providing the first high-quality evidence in favor of adding an immunosuppressive regimen to GC therapy in the management of this condition [33]. GCs plus either cyclophosphamide or mycophenolate mofetil had similar efficacy in reducing proteinuria after six months [67] and small studies reported response rates of 40–60% in patients treated with a combination of glucocorticoids and azathioprine [55], oral or intravenous cyclophosphamide [33], mycophenolate mofetil [55], calcineurin inhibitors [68] and rituximab [69].

In an Asian cohort, the combination of glucocorticoids, tacrolimus, and low-dose mycophenolate mofetil—known as the “multitarget therapy”—achieved a significantly higher complete remission rate in pMLN (33.1%) compared to glucocorticoids combined with high-dose cyclophosphamide followed by azathioprine (7.8%) [70].

In the recent phase 3 AURORA trial, 25 pMLN patients (14%) treated with voclosporin showed that the addition of voclosporin to glucocorticoids and low-dose mycophenolate mofetil resulted in a faster, though not statistically significant, proteinuria reduction compared to the control group (median time of 3.6 months vs. 8.3 months, *p* > 0.05) [71].

A post hoc analysis of the BLISS-LN trial suggested that belimumab provided less benefit in patients with nephrotic-range proteinuria compared to those with less severe proteinuria, though it may still help reduce the incidence of adverse kidney outcomes. Moreover, the benefits of belimumab were primarily seen in patients with proliferative features at kidney biopsy, with no significant effect observed in pMLN patients [72,73].

Moreover, an anti-CD20 type II engineered humanized monoclonal antibody called obinutuzumab has been recently evaluated as an add-on therapy in the phase 3 REGENCY trial, and was shown to be superior to the standard of care in the achievement of a complete renal response at 76 weeks. However, the study only included proliferative LN classes, with or without concurrent class V. Notably, the benefit of obinutuzumab was particularly evident in patients with mixed LN in whom the rate of complete renal response at week 76 was 38% versus 24% with placebo (95% CI, 6.3–44.7). Unfortunately, patients with pMLN were not included [74].

The optimal maintenance regimen for patients with pMLN remains a matter of debate. Existing guidelines do not specify a one-size-fits-all duration, but they emphasize individualization based on relapse risk, disease severity, side-effect profiles, and patient factors. In a recent cohort, 38 pMLN patients exhibited a 20-month shorter interval between renal response and immunosuppressive discontinuation compared to patients with proliferative classes. Of note, pMLN was associated with a minimal rate of post immunosuppression discontinuation flares and superior long-term outcomes, consistent with the findings of Zen et al. [75,76]. However, detailed analysis comparing baseline characteristics and induction regimens of pMLN vs. proliferative/mixed forms were not available and the results should be interpreted with caution. Further studies should examine the validity of these results, which could lead to reduced exposure to immunosuppression in histological subgroups.

## 3. Current Management and Future Perspectives

The most recent ACR guidelines for lupus nephritis management take into account the latest trial results and advocate for a similar triple treatment approach—including corticosteroids, mycophenolate, and calcineurin inhibitors—both in case of proliferative/mixed LN and of pMLN [66].

This paradigm shift challenges the traditional view of pMLN as a relatively benign condition and underscores the necessity of equally intensive treatment strategies. There are several unmet needs in the management of pMLN. One important area for future research is the investigation of molecular and genetic mechanisms that differentiate pure MLN from mixed and proliferative lupus nephritis, which could provide valuable insights into their distinct behaviors and clinical trajectories. Furthermore, there is an increasing need to evaluate both histological and non-histological factors, paving the way toward an ideal treatment approach tailored to the specific patient. Finally, conducting randomized controlled trials to determine the effectiveness of emerging therapies is crucial for improving the management of various LN subtypes. Moving forward, therapeutic decisions must be grounded in a personalized approach that integrates both clinical presentation and histopathological findings, enabling more accurate risk stratification and targeted disease management.

## Figures and Tables

**Table 1:** Modified ISN/RPS Classification of Lupus Nephritis (2018) [6,16].

Class	Name	Definition	Comments
I	Minimal mesangial lupus nephritis	Normal by LM with mesangial deposits by IF or EM	May have other features such as podocytopathy or tubulointerstitial disease (beware of unsampled class III).
II	Mesangial proliferative lupus nephritis	Purely mesangial hypercellularity by LM with mesangial deposits by IF; may be rare subepithelial or subendothelial deposits by IF or EM (not by LM).	May have other features such as podocytopathy, tubulointerstitial disease (beware of unsampled class III), or thrombotic microangiopathy.
III	Focal lupus nephritis	Active or inactive segmental or global endocapillary and/or extracapillary glomerulonephritis by LM in <50% of glomeruli; usually shows subendothelial deposits.	Active (A) & chronic (C) lesions defined in Modified NIH Activity & Chronicity Scoring System; replaces A & C designation.
IV	Diffuse lupus nephritis	Active or inactive endocapillary and/or extracapillary glomerulonephritis by LM in ≥50% of glomeruli.	Omitted segmental (S) & global (G) designations due to lack of reproducibility & clinical correlation; A & C lesions as defined in Modified NIH Activity & Chronicity Scoring System.
V	Membranous lupus nephritis	Global or segmental granular subepithelial deposits along GBM by LM & IF or EM; if class III or IV is present, needs to be in >50% of capillaries or >50% of glomeruli; ±mesangial alterations.	May occur with class III or IV, which are designated class III/V or class IV/V, respectively.
VI	Advanced sclerosing lupus nephritis	≥90% of glomerular sclerosis without residual activity.	Should be eliminated or reevaluated due to the recognizability of globally sclerotic glomeruli resulting from preceding lupus nephritis active lesions versus nonspecific global sclerosis associated with other factors (i.e., aging, hypertension, or healed TMA lesions).

Adapted from: Colvin, Robert, B. and Anthony Chang. Diagnostic Pathology: Kidney Diseases. Available from: Elsevier eBooks+, (3rd Edition). Elsevier—OHCE, 2019 [20]. LM, Light Microscopy; IF, Immunofluorescence; EM, Electron Microscopy; GBM, Glomerular Basement Membrane; TMA, Thrombotic microangiopathy.

**Table 2:** Modified NIH Lupus Nephritis Activity and Chronicity Scoring System [6,17].

NIH Activity Index	Definition	Score
Endocapillary hypercellularity (% glomeruli)	None (0), <25% (1+), 25–50% (2+), >50% (3+) of glomeruli	0–3
Neutrophils/karyorrhexis (% glomeruli)	None (0), <25% (1+), 25–50% (2+), >50% (3+) of glomeruli	0–3 × 2
Fibrinoid necrosis (% glomeruli)	None (0), <25% (1+), 25–50% (2+), >50% (3+) of glomeruli	0–3 × 2
Wire loops or hyaline thrombi (% glomeruli)	None (0), <25% (1+), 25–50% (2+), >50% (3+) of glomeruli	0–3
Cellular or fibrocellular crescents (% glomeruli)	None (0), <25% (1+), 25–50% (2+), >50% (3+) of glomeruli	0–3 × 2
Interstitial inflammation (% cortex)	None (0), <25% (1+), 25–50% (2+), >50% (3+) of cortex	0–3
Total (Activity Index):	0–24	
NIH Chronicity Index	Definition	Score
Glomerulosclerosis score, global and/or segmental (% glomeruli)	None (0), <25% (1+), 25–50% (2+), >50% (3+) of glomeruli	0–3
Fibrous crescents (% glomeruli)	None (0), <25% (1+), 25–50% (2+), >50% (3+) of glomeruli	0–3
Tubular atrophy (% cortex)	None (0), <25% (1+), 25–50% (2+), >50% (3+) of cortex	0–3
Interstitial fibrosis (% cortex)	None (0), <25% (1+), 25–50% (2+), >50% (3+) of cortex	0–3
Total (Chronicity Index):	0–12	

Note: Scores are summed. Activity scores generally decrease with treatment, while chronicity scores persist or increase. Adapted from: Colvin, Robert, B. and Anthony Chang. Diagnostic Pathology: Kidney Diseases. Available from: Elsevier eBooks+, (3rd Edition). Elsevier—OHCE, 2019 [20].

**Table 3:** Main studies examining long-term outcomes and prognosis of pure MLN.

	N °	Kidney Biopsy Histology	Race	Year	Follow-Up (yr)	Main Outcomes
Kapsia et al. [59]	27	Pure Class V *	Caucasian	2022	4.7	22% relapsed, no patient progressed to kidney failure
Kang et al. [54]	50	Classes V/II + V * (non-proliferative)	Asian	2022	8.6	At last follow-up: 29% had eGFR < 60 mL/min/1.73 m^2^. Low eGFR levels at 6 months were associated with poor renal outcomes.
Silva-Fernandez et al. [31]	150	Pure class V *	Caucasian	2017	7.6	20% showed impaired eGFR at diagnosis. At last observation: 5% kidney failure, 6% death.
Mejía-Vilet et al. [55]	60	Pure Class V *	Caucasian	2016	4.3	38.3% showed impaired eGFR at diagnosis; at last observation: 3.3% kidney failure, 5% death.
Okpechi et al. [56]	42	Pure Class V *	African American	2012	NA	26.2% of patients reached the composite endpoint (death, end-stage renal failure, or persistent doubling of serum creatinine).
Sun et al. [57]	100	Pure Class V *	Asian	2008	6.4	29.9% relapsed, 21 patients re-biopsied after 33 months: 8 (38.1%) transformed (5 patients transformed to class V + class IV, 2 patients in class V + III, and 1 patient in class VI). Patient survival at 5 and 10 years: 98%. Renal survival at 5 and 10 years: 96.1% and 92.7%
Pasten et al. [58]	33	Va/Vb °	Caucasian	2005	5.3	At last observation: 33% death, 25% had CrCl < 15 mL/min at 5 years
Mok et al. [32,60]	38	Va/Vb °	Asian	2004 and 2009 (long term outcome study)	7.5 and 12	2004: 67% complete remission, 19% renal flares, 13% had Cr/Cl decline by 20%. 2009: no patient progressed to kidney failure
Mercadal et al. [19]	66	Va/Vb °	48% Caucasian, 47% African American	2002	6.9	49% relapsed; renal survival at 5 and at 10 years: 97%, and 88%; kidney failure rate: 9%.

° WHO [16]; * ISN/RPS 2003 [6,16]; CrCl, Creatinine Clearance; eGFR, estimated Glomerular Filtration Rate; pMLN, pure Membranous Lupus Nephritis.

**Table 4:** Main studies comparing pure MLN to proliferative/mixed histological classes.

	N °	Comparison	Pure Membranous	Mixed Classes	Pure Proliferative	Race	Year	Follow-Up (yr)	Main Outcomes
Kharouf et al. [22]	215	Class V vs. Classes III or IV vs. classes VII + V/IV + V *	51	44	120	Australian	2024	8	No differences in complete renal response, proteinuria recovery. PLN vs. pMLN/mixed LN: trend towards worse long-term outcomes
Farinha et al. [61]	260	Class V vs. Classes III or IV *	47	(10 ^)	203	Caucasian	2024	8	pMLN: lower creatinine at onset PLN: low C3–C4, higher anti-DNA positivity. At last observation: CKD: 17% PLN vs. 7% pMLN; ESKD: 4% vs. 2%, mortality: 7% vs. 2%
Moroni et al. [21]	103	Class V vs. Classes III + V/IV + V *	67	36	NA	Caucasian	2012	13	Mixed classes: more frequent nephrotic syndrome at onset, low C3-C4, anti-DNA positivity, higher activity-chronicity indexes. No differences in remission (94.5 vs. 94.0%) and kidney survival (85.8 vs. 86.0%) at 10 years between groups
Sloan et al. [18]	79	WHO Va/Vb vs. Vc/Vd °	36	43	NA	American	1996	4	Va/Vb better prognosis than Vc/Vd: 10-y renal survival 72% Va/Vb vs. 20–49% Vc/Vd

° WHO [16]; * ISN/RPS 2003 [6,16]; ^ not involved in the comparison; Anti-dsDNA, autoantibodies anti-double strand DNA; PLN, Proliferative Lupus Nephritis; MLN, Membranous Lupus Nephritis; CKD, Chronic Kidney Disease; ESKD, End-Stage Kidney Disease.

## Data Availability

The data presented in this study are available on request from the corresponding author due to privacy reasons.
